# Association of physical activity level and all-cause mortality among stroke survivors: evidence from NHANES 2007–2018

**DOI:** 10.1265/ehpm.24-00322

**Published:** 2025-04-22

**Authors:** Fude Liu, Xiangning Han, Yawen Cheng, Ning Zhu, Shiliang Jiang, Jiahao Li, Jin Zhao, Guogang Luo

**Affiliations:** 1 Department of Neurology, The First Affiliated Hospital of Xi’an Jiaotong University, Xi’an, China; 2 Department of Nephrology, Xijing Hospital, Fourth Military Medical University, Xi’an, China

**Keywords:** Physical activity, Stroke, All-cause mortality

## Abstract

**Background:**

Post-stroke disability diminishes the physical activity (PA) level of survivors, potentially affecting their long-term prognosis. This study endeavors to explore the correlation between daily PA level and the all-cause mortality in patients with a history of stoke in the United States.

**Methods:**

Data of stroke survivors were sourced from the National Health and Nutritional Examination Survey (NHANES) 2007–2018. The population was stratified into three groups based on their PA level. Kaplan-Meier method with log-rank tests for significance was used for survival analysis. Weighted Cox proportional hazards regression models were employed to estimate the hazard ratios (HRs) for all-cause mortality. Subgroup analysis was conducted to strengthen the results.

**Results:**

A total of 1395 participants were recruited, comprising 679 males and 716 females, with a median age of 68 years. Based on their PA levels, 779 individuals were classified as inactive, 156 as insufficiently active, and 460 as sufficiently active. Following a median observation period of 59 months, there were 476 recorded deaths, with 349, 47, and 80 cases in the three respective groups. Compared to the inactive group, the HRs and 95% confidence intervals (CIs) for all-cause mortality in participants who were insufficiently active and sufficiently active were 0.58 (0.40, 0.84) and 0.47 (0.33, 0.67), respectively. The Kaplan-Meier curve revealed a significant difference in overall survival between the three groups, as confirmed by the log-rank test (P < 0.0001). Subgroup analysis further validated our results and demonstrated that the protective impact of PA on stroke prognosis varies according to distinct characteristics.

**Conclusions:**

The results indicate that increased levels of PA are associated with a protective effect on long-term mortality among stroke survivors. Further prospective longitudinal studies are necessary to elucidate the optional PA level and special exercise guideline targeting this population.

## 1. Introduction

Stroke, a widespread neurological condition marked by significant morbidity and mortality, ranks as the second leading cause of death globally, preceded only by ischemic heart disease [1, 2]. Since the start of the decade in 2010, the worldwide incidence of stroke has been rising at a concerning average annual rate of 2.5%, resulting in an increase of approximately 270,000 new cases each year [3]. In 2019, this upward trend culminated in a staggering 12.2 million new stroke diagnoses, accompanied by 6.6 million stroke-related deaths, leading to a total of 101 million individuals 101 million individuals globally who have a history of stroke [4]. Worryingly, predictions indicate that a quarter of the world’s population is at risk of experiencing a stroke, with an individual’s lifetime risk climbing by almost 9% over three decades [2]. Although progress has been made in improving the survival rates of stroke patients, the high recurrence rate remains a significant challenge, posing a serious threat to those with a history of stroke. This highlights the critical need for persistent medical research and interventions to alleviate the impact of stroke and enhance patient outcomes.

Moreover, stroke carries a significant risk of disability, with more than half of survivors experiencing disability, resulting in decreased physical activity (PA) among them [5]. For numerous stroke survivors, the initial acute stroke event marks the commencement of a persistent battle with disability and consequent physical impairment [6]. The lack of activity not only impairs daily functioning but also heightens the chances of recurrent stroke and mortality [7]. Additionally, stroke survivors also face increased risks of cardiovascular disease and diabetes due to insufficient daily activity [8]. Conversely, sustaining relative high levels of PA exerts protective effects, guarding against a range of chronic conditions including diabetes, cardiovascular diseases, and cancer, while diminishing the overall mortality rate [9]. Similarly, PA may possess neuroprotective properties and reduce the risk factors associated with stroke [10]. Post-stroke exercise has also shown to be an effective preventive strategy for reducing disability and improve long-term outcome among individuals with stroke [11, 12]. The guideline for primary care of patients after stroke also suggest aerobic exercise as important approach for cardiovascular health and secondary stroke prevention, regardless of specific rehabilitation needs [13]. Yet, the effect of the daily PA level on the prognosis of stroke remains still inconclusive, and the optimal level of PA and its benefits in stroke remain unclear.

This study aims to investigate the relationship between daily PA levels and the risk of all-cause mortality among U.S. adults who have experienced a stroke, using a nationally representative sample of the American population.

## 2. Methods

### 2.1 Data source and study population

**Fig. 1:**
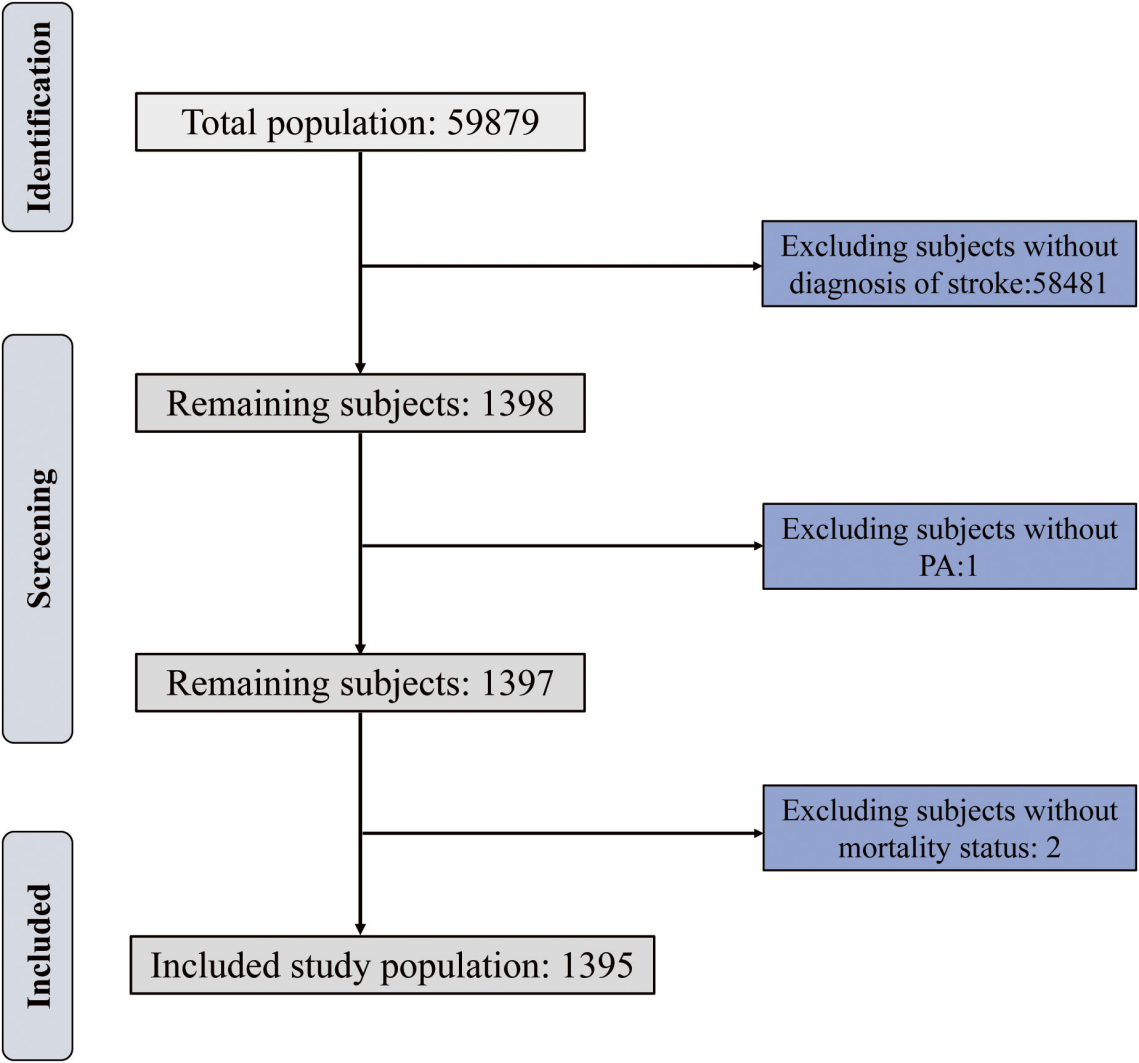
Diagram illustrating the selection process of the study participants.

We obtain the data from the National Health and Nutritional Examination Survey (NHANES), a continuous cross-sectional survey in the U.S., with stratified, multistage probability design to assess the health and nutritional status of the general population. Demographics data, dietary data, examination data, laboratory data, and questionnaire data are collected and released every two years. The study protocols received approval from the Ethics Review Board of the National Center for Health Statistics, with participants providing written informed consent. Since the PA questionary data remain consistent across 2007–2018, we construct an observational cohort of stroke patients by combing these six NHANES cycles baseline data with corresponding National Death Index (NDI) mortality data. Individuals under 18 years old and with missing information on PA or death status were excluded. The population was stratified into three groups based on their PA level. The detail of participants selection process is presented in Fig. 1.

### 2.2 Definition of stroke

Consistent with prior research, a stroke was identified based on self-reported past diagnoses made by a doctor during in-person interviews [14]. Participants who answered “yes” to the question, “Has a doctor or other health professional ever told you that you had a stroke?” were categorized as stroke survivors.

### 2.3 Calculation of MET and PA level categories

The Metabolic Equivalent of Task (MET) is a metric that determines the oxygen consumption needed to maintain resting metabolism while seated. It serves as a crucial indicator of relative energy metabolism levels and exercise intensity. In NHANES, physical activity was evaluated using the Global Physical Activity Questionnaire (GPAQ). The NHANES provides suggested MET Scores for specific physical activity patterns, with 8.0 for vigorous work-related activity or vigorous leisure-time physical activity, and 4.0 for moderate work-related activity, moderate leisure-time physical activity, and walking or bicycling for transportation. To measure PA levels, the weekly MET minutes (MET-min/week) were computed using the following formulas: MET-min/week = MET value × Frequency per week × Duration per session. Then, in accordance with the Physical Activity Guidelines for Americans, PA levels were divided into three categories: (1) inactive (0 MET-min/week), insufficiently active (1–599 MET-min/week), and sufficiently active (≥600 MET-min/week).

### 2.4 Mortality ascertainment

Utilizing precise sequence number matching for each respondent, mortality information was acquired from the publicly accessible NHANES mortality files, which were sourced from the National Death Index (NDI). In cases where no match was found in the NDI, it was presumed that the individual was still alive. The duration from the NHANES household interview date until the last confirmed date of survival status was computed as the event timeline for each participant. The follow-up period for participants ended on December 31, 2019.

### 2.5 Covariates assessment

This study considered multiple factors, including key demographic details, physical assessment findings, and concurrent health conditions. Table 1 presents variables such as gender, ethnicity, body mass index (BMI), family income relative to the poverty threshold, and educational attainment. Alcohol intake was categorized into three groups: non-drinkers, moderate drinkers (defined as one alcoholic beverage daily for females and one to two drinks daily for males), and excessive drinkers (more than two drinks daily for females and over three drinks daily for males). Smoking habits were classified based on self-reported information into non-smokers, ex-smokers, and active smokers. The presence of diabetes, hypertension, and abnormal blood lipid levels was determined through a doctor’s diagnosis or laboratory tests following the relevant diagnostic guidelines.

**Table 1:** Baseline characteristics of included participants.

**Characteristic**	**Total**	**Physical activity**			***P value***

		**Inactive**	**Insufficiently active**	**Sufficiently active**	
**Participants, n**	1395	779	156	460	
**Gender, %**					0.041
Male	679 (44.1%)	355 (40.9%)	72 (39.0%)	252 (50.1%)	
Female	716 (55.9%)	424 (59.1%)	84 (61.0%)	208 (49.9%)	
**Age, years**	67.63 (12.77)	67.63 (12.77)	66.74 (12.89)	60.46 (13.95)	<0.001
**Race or ethnicity, %**					0.289
Non-Hispanic White	541 (50.4%)	311 (50.0%)	65 (56.9%)	165 (49.2%)	
Non-Hispanic Black	527 (33.8%)	285 (34.6%)	61 (32.3%)	181 (33.1%)	
Mexican American or Hispanic	215 (7.9%)	129 (8.8%)	17 (4.3%)	69 (7.8%)	
Other	112 (7.8%)	54 (6.6%)	13 (6.4%)	45 (9.9%)	
**BMI, kg/m^2^, %**					0.012
<25.0	316 (22.7%)	158 (20.5%)	45 (31.5%)	113 (23.4%)	
25.0–29.9	391 (27.3%)	213 (25.4%)	37 (24.8%)	141 (30.6%)	
≥30.0	538 (40.0%)	290 (40.4%)	65 (40.3%)	183 (39.2%)	
Missing data	150 (10.0%)	118 (13.6%)	9 (3.4%)	23 (6.8%)	
**Education level, %**					<0.001
Less than high school	483 (26.8%)	322 (33.5%)	51 (23.6%)	110 (18.4%)	
High school	380 (30.4%)	201 (29.3%)	41 (34.8%)	138 (30.7%)	
College or higher	529 (42.6%)	253 (37.0%)	64 (41.6%)	212 (50.9%)	
Missing data	3 (0.1%)	3 (0.2%)	0 (0%)	0 (0%)	
**Ratio of family income to poverty, %**					
≤1.30	523 (29.7%)	292 (31.5%)	68 (33.6%)	163 (26%)	0.057
1.31–3.50	517 (39.4%)	296 (41.9%)	48 (36.7%)	173 (36.6%)	
>3.50	229 (22.4%)	114 (17.6%)	27 (22.0%)	88 (29.1%)	
Missing data	126 (8.6%)	77 (9.0%)	13 (7.6%)	36 (8.4%)	
**Alcohol, %**					0.001
None	358 (23.5%)	220 (26.1%)	46 (27.5%)	92 (18.7%)	
Moderate	277 (23.2%)	120 (18.6%)	37 (26.8%)	120 (28.7%)	
Heavy	207 (16.7%)	91 (13.1%)	18 (19.6%)	98 (21.1%)	
Missing data	553 (36.5%)	348 (42.2%)	55 (26.1%)	150 (31.5%)	
**Smoke, %**					0.29
Nonsmoker	564 (41.9%)	325 (42.6%)	65 (41.0%)	174 (41.2%)	
Former smoker	497 (34.1%)	293 (36.0%)	58 (37.8%)	146(30.3%)	
Current smoker	334 (24.0%)	161 (21.4%)	33 (21.2%)	140 (28.4%)	
Missing data	0 (0%)	0 (0%)	0 (0%)	0 (0%)	
**Diabetes, %**	491 (32.3%)	312 (38.1%)	56 (28.6%)	123 (24.8%)	0.003
**Hypertension, %**	1166 (81.4%)	668 (84%)	136 (88.2%)	362 (75.8%)	0.02
**Dyslipidemia, %**	934 (67.4%)	522 (68.8%)	109 (67.1%)	303 (65.6%)	0.16
**MET**	1838 (4287.86)	0 (0)	293 (147.78)	4884 (5915.51)	<0.001
**All-cause mortality, n (%)**	476 (34.1%)	349 (44.8%)	47 (30.13%)	80 (17.39%)	
**Follow time, months**	59 (31, 98)	57 (29, 92)	63 (36, 101)	59 (32, 104)	

Values are weighted mean (SE) for continuous variables or numbers (weighted %) for categorical variables. BMI, body mass index; MET, Metabolic Equivalent of Task.

### 2.6 Statistical analysis

To accommodate the intricate sampling framework of NHANES, we applied suitable sample weights, ensuring that our findings accurately reflected the broader U.S. population. Continuous variables, excluding age and MET, were categorized, with missing values being treated as a separate category for analysis. We computed descriptive statistics within each group, relying on median (interquartile range, IQR) for continuous data and proportions for categorical data. To assess baseline characteristic differences among groups, we employed the Mann-Whitney U-test for continuous variables and weighted chi-square tests for categorical ones. Survival trends were explored using the Kaplan-Meier method, with significance determined via log-rank tests. For a comprehensive understanding of all-cause mortality risk, we utilized weighted multivariable Cox proportional hazards regression models. Our analysis progressed through four models, adjusting for potential confounders at each step. Model 1 remained unadjusted, while Model 2 considered age, sex, and race. Model 3 enhanced Model 2 by incorporating additional adjustments for lifestyle and physical variables, including alcohol consumption, smoking habits, BMI, and socioeconomic measures such as the family income-to-poverty ratio and educational attainment. Finally, Model 4 encompassed all variables from Model 3 and added adjustments for health conditions including diabetes, hypertension, and dyslipidemia. Furthermore, we carried out stratified analyses to explore potential modifiers and confirm the findings. All statistical computations were executed utilizing R software, adopting a significance level of P < 0.05 as the criterion for statistical significance.

### 2.7 Ethics approval and consent to participate

This study did not necessitate supplementary ethical clearance or permissions, as it employed data from the NHANES program, which had already received approval from the Ethics Committee of National Center for Health Statistics (NCHS). The protocol numbers corresponding to the NHANES survey cycles from 2007 to 2018 are as follows: #2005-06, #2011-17, and #2018-01. Moreover, all participants had provided written consent.

## 3. Results

### 3.1 Patient characteristics

In our study, a cohort of 1395 participants were recruited, comprising 679 males and 716 females, with a median age of 68 years. Categorized based on their PA levels, the distribution was as follows: 779 individuals were classified as inactive, 156 as insufficiently active, and 460 as sufficiently active. Following a median observation period of 59 months, there were 476 recorded deaths, with 349, 47, and 80 cases in the three respective groups. The baseline characteristics revealed significant differences in the distribution of gender, age, BMI, education level, alcohol consumption habits, and the prevalence of diabetes and hypertension (*P*-values <0.05). The demographic and clinical characteristics of the study population are summarized in Table 1, providing a baseline profile of the patients under investigation.

### 3.2 Kaplan-Meier survival curve analysis

**Fig. 2:**
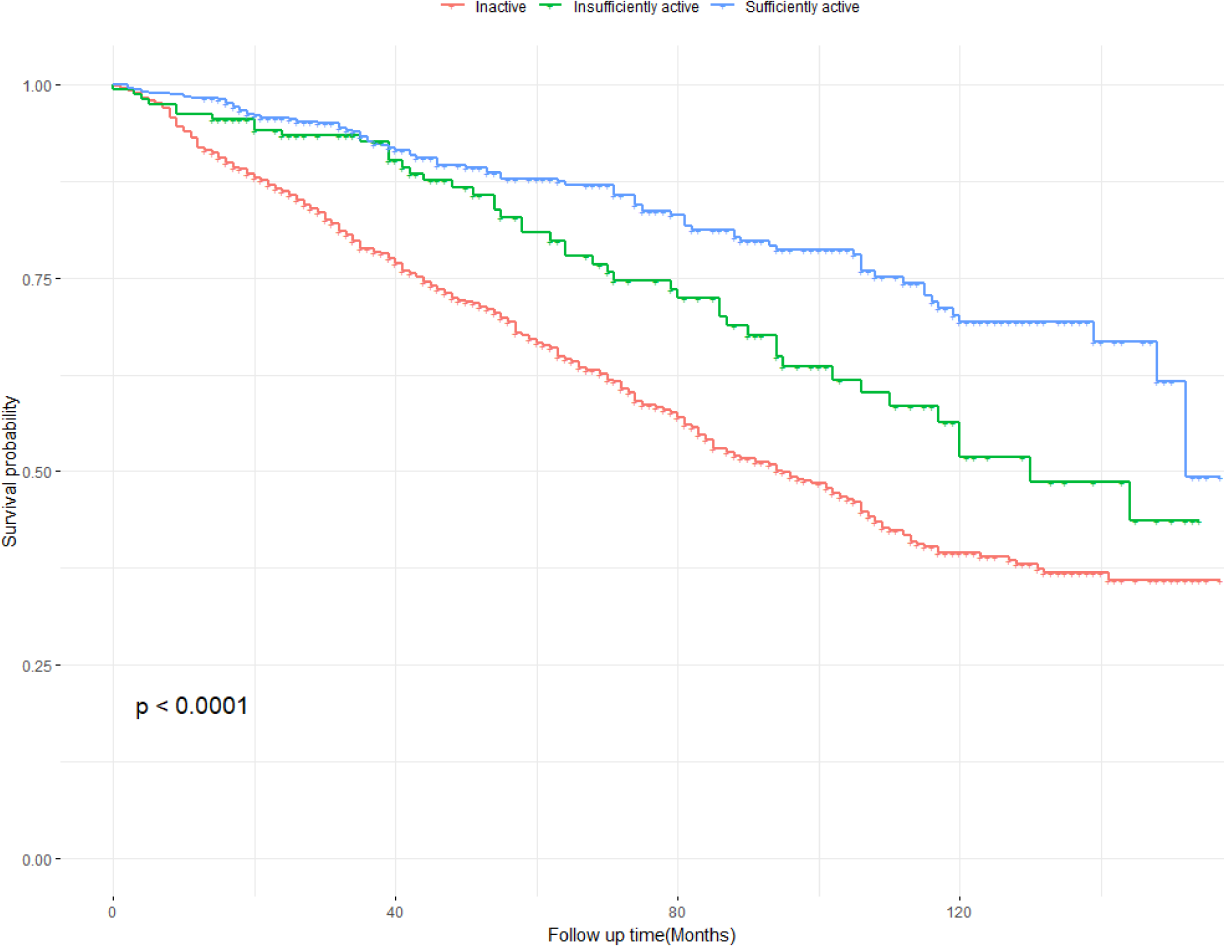
Kaplan-Meier curves of the survival rate with different PA group. PA, physical activity

The Kaplan-Meier curve demonstrated a significant difference in survival probability among the inactive (Fig. 2), insufficiently active, and sufficiently active groups for all-cause mortality (log-rank test, p < 0.001). Specifically, the sufficiently active group exhibited significantly higher survival rates compared to both the inactive and insufficiently active groups, indicating that maintaining an adequate level of physical activity positively impacts survival outcomes.

### 3.3 Relationship between PA and all-cause mortality

Table 2 shows the multivariate-adjusted hazard ratios (HRs) with 95% confidence intervals (CI) based on PA as a categorical variable. In the unadjusted model (Model 1), the HRs and 95% CIs for all-cause mortality in participants with insufficient activity compared to those who were inactive (reference) were 0.59 (0.43, 0.80), and for participants with sufficient activity, the HR was 0.33 (0.24, 0.45). Similarly, in model 4, the HRs and 95% CIs for all-cause mortality in participants with insufficient and sufficient activity were 0.58 (0.40, 0.84) and 0.47 (0.33, 0.67), respectively.

**Table 2:** Associations of PA with all-cause mortality among US stroke survivors.

	**No. of Events**	**HR (95% CI)**			

		**Model 1**	**Model 2**	**Model 3**	**Model 4**
**All-cause mortality**					
Inactive	349/779	1.0(reference)	1.0(reference)	1.0(reference)	1.0(reference)
Insufficiently active	47/156	0.59 (0.43, 0.80)	0.59 (0.42, 0.84)	0.59 (0.41, 0.85)	0.58 (0.40, 0.84)
Sufficiently active	80/460	0.33 (0.24, 0.45)	0.45 (0.33, 0.61)	0.47 (0.33, 0.67)	0.47 (0.33, 0.67)

Values are n or weighted HR (95% CI). Model 1 is unadjusted; Model 2 is adjusted for: age, gender and race; Model 3 is adjusted for: Model 2 + alcohol intake, smoking status, BMI, ratio of family income to poverty, and education level; Model 4 is adjusted for: Model 3 + diabetes, hypertension, and dyslipidemia. PA, physical activity; HR, hazard ratio; CI, confidence interval; BMI, body mass index.

### 3.4 Subgroup analysis

Table 3 displays subgroup analyses that corroborate our preliminary findings across various subgroups. The protective effect of physical activity (PA) on mortality risk is more pronounced in females (HR 0.41, 95% CI 0.27–0.64), Non-Hispanic Whites (HR 0.41, 95% CI 0.22–0.77), those with a high family income-to-poverty ratio (HR 0.37, 95% CI 0.15–0.88), current smokers (HR 0.21, 95% CI 0.10–0.43), non-alcohol consumers (HR 0.24, 95% CI 0.11–0.51), individuals with a BMI ≤25 (HR 0.26, 95% CI 0.14–0.49), those with a high education level (HR 0.31, 95% CI 0.19–0.51), and hypertensive individuals (HR 0.44, 95% CI 0.30–0.66). However, comorbidities such as diabetes (diabetes, HR 0.77, 95% CI 0.42–1.40; non-diabetes, HR 0.45, 95% CI 0.23–0.85) and dyslipidemia (HR 0.63, 95% CI 0.41–0.97) appear to diminish the protective effect.

**Table 3:** Subgroup analysis of associations of PA with all-cause mortality among US stroke survivors.

**Subgroup**	**HR (95% CI) for All-cause mortality**	

	**Insufficiently active**	**Sufficiently active**
**Age, years**		
≤65	0.58 (0.39, 0.85)	0.35 (0.24, 0.51)
>65	0.50 (0.34, 0.73)	0.35 (0.24, 0.52)
**Sex**		
Male	0.58 (0.35, 0.95)	0.45 (0.29, 0.71)
Female	0.53 (0.30, 0.93)	0.41 (0.27, 0.64)
**Race**		
Non-Hispanic White	0.62 (0.37, 1.06)	0.41 (0.22, 0.77)
Non-Hispanic Black	0.42 (0.23, 0.77)	0.49 (0.29, 0.84)
**Ratio of family income to poverty**		
≤1.3	0.61 (0.35, 1.04)	0.54 (0.33, 0.89)
1.3–3.5	0.37 (0.17, 0.78)	0.51 (0.31, 0.86)
>3.5	0.38 (0.18, 0.83)	0.37 (0.15, 0.88)
**Smoking status**		
Nonsmoker	0.67 (0.41, 1.08)	0.45 (0.26, 0.78)
Former smoker	0.54 (0.28, 1.03)	0.48 (0.28, 0.80)
Current smoker	0.31 (0.13, 0.75)	0.21 (0.10, 0.43)
**Alcohol intake**		
None	0.61 (0.36, 1.04)	0.24 (0.11, 0.51)
Moderate	0.51 (0.20, 1.32)	0.69 (0.35, 1.38)
Heavy	0.36 (0.10, 1.35)	0.38 (0.15, 0.94)
**BMI**		
≤25	0.29 (0.15, 0.54)	0.26 (0.14, 0.49)
25–30	0.88 (0.33, 2.37)	0.47 (0.28, 0.80)
>30	0.86 (0.46, 1.61)	0.61 (0.29, 1.26)
**Education level**		
Less than high school	0.72 (0.40, 1.27)	0.45 (0.24, 0.82)
High school	0.61 (0.26, 1.39)	0.69 (0.41, 1.16)
College or higher	0.37 (0.16, 0.87)	0.31 (0.19, 0.51)
**Diabetes**		
Yes	0.77 (0.42, 1.40)	0.59 (0.28, 1.23)
No	0.45 (0.23, 0.85)	0.33 (0.22, 0.50)
**Hypertension**		
Yes	0.57 (0.38, 0.87)	0.44 (0.30, 0.66)
No	0.85 (0.27, 2.71)	0.87 (0.37, 2.04)
**Dyslipidemia**		
Yes	0.63 (0.41, 0.97)	0.53 (0.35, 0.79)
No	0.45 (0.19, 1.02)	0.26 (0.12, 0.55)

PA, physical activity; HR, hazard ratio; CI, confidence interval; BMI, body mass index.

## 4. Discussion

As lifestyles evolve, the influence of PA on health is garnering heightened interest. As stroke ranks as the foremost cause of chronic disability in the United States [15], the level of PA in stroke patients significantly influences their rehabilitation and prognosis [10]. In this observational study involving 13,95 stroke survivals, an investigation was undertaken to delve into the connection between the levels of PA and all-cause mortality. The study revealed a significant inverse correlation where higher PA levels among stroke survivors were associated with a reduced risk of mortality. This protective effect remained significant even after adjusting for potential confounding factors. The findings of this study further reinforce and underscore the significance of PA in stroke prognosis, offering robust evidence for the healthcare providers to prioritize PA-based intervention as a key component of post-stroke care, ultimately improving outcomes for stroke survivors.

There is a growing emphasis on understanding the relationship between PA with stroke. In the study exploring this relationship, Cowan LT et al. indicated that increasing PA levels can significantly reduce the risk of stroke in the middle-aged American population [16]. Hung SH et al. also found that increasing the duration and intensity of physical exercise in daily life is associated with reducing the severity of stroke [17]. For stroke survivors, Dongni et al. discovered that increased PA was associated with functional recovery 6 months after stroke [18]. A cohort study conducted in Canadian Community also found PA was associated with a reduced risk of all-cause mortality among young stroke survivals in a dose-responsive manner [19]. Our findings further validate that increased levels of PA are associated with improved long-term survival rates among stroke survivors.

The factors contributing to the poor prognosis of stroke patients with reduced PA levels are complex and multifaceted. Besides the disability directly stemming from stroke, the unique circumstances surrounding stroke patients further amplify the decline in PA. Our research has illuminated that the preponderance of stroke patients are elderly, with a median age of 68, and they frequently grapple with a multitude of chronic diseases, notably hypertension (81.4%), diabetes (32.3%), and dyslipidemia (67.4%). These comorbidities may collectively impair cardiorespiratory fitness, cognitive ability, and physical motor function, thereby diminishing their capacity for PA. In return, lack of PA can often coexist with sedentary behavior (SB), creating a vicious cycle that intensifies the vulnerability to a wide spectrum of cardiovascular and cerebrovascular diseases [20–23]. This heightened risk not only augments the likelihood of future strokes but also undermines the prognosis and recovery outcomes of those already affected.

The potential mechanisms behind the beneficial outcomes of PA for stroke patients are multifaceted. Regular PA may contribute to enhanced cardiac function and vascular elasticity [24], reduced blood pressure [25], improved insulin sensitivity [26], decreased inflammation levels, and mitigated oxidative stress [27]. Additionally, increased PA has been demonstrated to facilitate the secretion of neurotrophic factors, promoting neural regeneration and repair [28], and enhancing neuroprotectivity and neuroplasticity after stroke [29]. Moreover, PA can mitigate cognitive decline in the elderly, which is essential for sustaining PA levels in older stroke patients. In summary, the beneficial effects of PA for stroke patients encompass both physiological and psychosocial aspects, especially for those with comorbidities of cardiovascular disease, metabolic disease, and cognitive impairment.

The link between PA and reduced mortality risk is modulated by diverse variables, including gender, age, BMI, educational attainment, and income status [30, 31]. In order to examine the influence of PA on stroke prognosis across diverse populations, we performed subgroup analyses. These analyses consistently demonstrated that enhancing physical exercise can diminish the mortality risk for stroke survivors, irrespective of their demographic attributes. The relationship between gender and PA in relation to mortality risk is a subject of considerable debate. Some research indicates that males experience a more substantial reduction in death risk from engaging in daily activities [31], while other studies suggest that females show a greater decrease in mortality risk through participation in PA [32, 33]. Consistent with the latter, regarding stroke survivals, we found females benefit more with reduced mortality risk. Moreover, numerous factors can influence the efficacy of exercise in enhancing the prognosis for stroke patients, including race, income, smoking status, alcohol habits, educational level, etc. Our study demonstrates that the demographic characteristics and clinical conditions of stroke survivals should be considered when developing PA intervention measures. It is important to note that the benefits of sufficiently activity are limited for patients with diabetes and hyperlipidemia, compared to those without comorbidity. This indicates a need to concurrently intensify the treatment of comorbidities while increasing PA levels to maximize overall benefits.

Our study possesses noteworthy strengths across various dimensions. Firstly, the substantial sample size employed ensures the robustness and applicability of our findings, making the conclusions more reliable and generalizable. Secondly, by carefully choosing and modifying covariates, we have effectively minimized the confounding effects of non-research factors on data interpretation, ensuring a clearer understanding of the underlying relationships. Additionally, we performed a precise patient subgrouping and data analysis, which revealed intricate and nuanced relationships within distinct individual characteristics. However, our study has several limitations that warrant careful consideration. First, as an observational analysis, our findings cannot establish causality between PA and mortality in stroke survivors. While our findings provide valuable insights into the potential association, they should be interpreted with caution. Intervention studies or randomized trials are essential to confirm the direct effects of PA on mortality. Second, the self-reported nature of PA and educational attainment in NHANES may introduce bias. Although NHANES employs standardized protocols to enhance data accuracy and studies comparing NHANES self-reported data have obtained valuable research findings [34, 35], biases inherent to questionnaire-based studies remain. For example, stroke survivors with cognitive impairments might inaccurately recall their activity levels [36], while healthier participants could overreport their exercise habits. These biases should be vigilantly noted when interpreting our conclusions. Third, while we adjusted for demographic and clinical confounders, critical factors such as nutritional status, social support networks, and mental health (e.g., post-stroke depression or anxiety) were not included in the analysis. These variables could independently influence both PA engagement and mortality risk, potentially skewing the observed associations [37–39]. We recommend that future research take these potential confounding factors into account to enhance the reliability and validity of the conclusion. Moreover, the generalizability of our findings is limited by the U.S.-centric NHANES cohort. Global variations in healthcare systems, socioeconomic disparities, and cultural attitudes towards PA may impact the applicability of our results in different regions. For instance, countries with universal healthcare systems might show distinct mortality patterns [40]. Therefore, validating our findings across diverse geographical settings is essential for a comprehensive understanding of the relationship between PA and mortality among stroke survivors worldwide. Finally, NHANES broadly classifies activity levels as moderate- and vigorous-intensity physical activities, including the frequency and duration of these activities, without specifying types (e.g., aerobic or resistance training). More detailed activity information would be beneficial for formulating more practical exercise guidelines. Future studies could leverage additional data sources or more granular measurement tools to supplement NHANES data and provide more detailed insights into specific activities and exercise intensity. Despite these limitations, our study provides valuable insights into the potential association between PA and mortality in stroke survivors. We hope that future research will address these limitations by incorporating more comprehensive data and considering additional confounding factors to further elucidate this relationship.

## 5. Conclusion

Increased PA significantly reduces long-term mortality in stroke patients. The prognosis improvement for various stroke populations differs with rising PA levels, necessitating the development of targeted, individualized exercise intervention plans tailored to the distinct characteristics of these populations. For patients with additional cardiovascular and metabolic diseases, active treatment of comorbidities should be integrated with their PA intervention. Future prospective studies should further explore the impact of diverse exercise interventions on both the physical function and mental health of stroke survivors, aiming to offer enhanced clinical guidance.

## Declarations

### Availability of data and material

The data relevant to my research is stored in the publicly accessible repository on the NHANES website (https://wwwn.cdc.gov/nchs/nhanes/Default.aspx).

### The use of AI

This manuscript was written entirely by the authors without the use of any artificial intelligence tools.
